# Inflammatory Cutaneous Diseases in Renal Transplant Recipients

**DOI:** 10.3390/ijms17081362

**Published:** 2016-08-19

**Authors:** Paola Savoia, Giovanni Cavaliere, Elisa Zavattaro, Federica Veronese, Paolo Fava

**Affiliations:** 1 Department of Health Sciences, “A. Avogadro” University of Eastern Piedmont, 28100 Novara, Italy; elisa.zavattaro@med.uniupo.it (E.Z.); federica.veronese@med.uniupo.it (F.V.); 2 Department of Medical Science, University of Turin, 10126 Turin, Italy; g.cavali_dr@yahoo.it (G.C.); fava_paolo@yahoo.it (P.F.)

**Keywords:** cutaneous diseases, inflammatory, kidney transplantation

## Abstract

Kidney transplant recipients frequently suffer from skin infections and malignancies, possibly due to the effects of long-term immunosuppressive therapy. While the relationships between immunosuppression and these pathological conditions have been widely investigated, little is known about the relative incidence and characteristics of inflammatory skin diseases in this type of patient. In this study, we analyze the incidence of a number of inflammatory cutaneous diseases in a cohort of patients who underwent kidney transplantation. Although our study shows a relatively low incidence of these pathologies in transplanted patients—in agreement with the general action of immunosuppressant therapies in reducing inflammation—we scored a different efficacy of the various immunosuppressive regimens on inflammatory and autoimmune skin diseases. This information can be key for designing immunosuppressive regimens and devising accurate follow-up protocols.

## 1. Introduction

Patients with chronic renal insufficiency often suffer from pathological conditions such as skin dryness, alopecia, skin discoloration, hair and nail abnormalities, as well as cutaneous diseases specifically related to kidney failure [1,2,3,4]. Usually, in the months immediately following transplantation, there is a progressive regression of these conditions, with a clinical and histopathological normalization of the skin [5]. However, other cutaneous diseases may progressively develop, which afflict more than half of renal transplant recipients, including infections to different body districts and non-melanoma skin cancers; these side effects are iatrogenic and develop as a consequence of the long-term immunosuppressive treatment. Notably, inflammatory diseases of the skin are rarely reported [6], possible as a consequence of the direct therapeutic effect of immunosuppressive therapy on these diseases. In the most recent Oxford series of kidney transplant recipients [6], the prevalence of psoriasis and atopic dermatitis was very low, around 1.5%, as also reported in other case series [7,8]. This is not surprising, since cyclosporine is commonly used in the treatment of psoriasis and other inflammatory dermatoses [9], while tacrolimus and mycofenolate are currently proposed for the treatment of atopic dermatitis [10].

Here, we describe the experience of our Clinical Units regarding the incidence and characteristics of various inflammatory skin diseases in a cohort of kidney transplant recipients, followed-up from 2009 to 2016.

## 2. Results

We analyzed the records obtained from a cohort of 610 renal transplant recipients (230 females, 380 males) followed-up at our center from January 2009 to April 2016. The median age was 60 years, while the mean age was 59. Clinical characteristics of these patients and the type of immunosuppressive regimen are summarized in Table 1, together with data about the prevalence of cutaneous diseases in this cohort. According to the treatment schedule in use at our center, the majority of patients (491, 81.5%) received an immunosuppressive regimen without mTOR inhibitors (mTOR-Is), whereas the remaining 19.5% (119 patients) received sirolimus or everolimus.

In our series, the most frequent dermatological diseases were skin infections, observed in 187 patients (30.7%), of which there were 72 virus-related infections (*Human Papilloma Virus-HPV*, *Varicella Zoster Virus-HZV*, *Herpes Simplex Virus-HSV type 1 and 2*), 57 cases of cutaneous mycosis (*Tinea unguium*, *Pytiriasis versicolor* and *Tinea corporis*), and 28 bacterial infections (folliculitis and impetigo), while 30 patients had concomitant viral and fungal infections.

A diagnosis of skin cancer during follow-up was made in 191 cases (31.3%); according to the histological type, we scored basal cell carcinomas (BCCs) in 84 patients, squamous cell carcinomas (SCCs) in 51, melanomas in 16, Kaposi’s Sarcoma in 17, cutaneous T-cell lymphomas (CTCLs) in four and other skin tumors in nine.

### 2.1. Inflammatory Diseases

Among the 610 kidney transplant recipients of our series, 88 showed an inflammatory skin disease (14.4%). Table 2 summarizes their clinical characteristics, together with data about the time and type of immunosuppressive regimen. Twenty-six patients were female (29.5%) and 62 were male (70.5%). The median age was 47 years, whereas no differences were found in the median duration of immunosuppression (9.1 years) and the follow-up duration with respect to the rest of our series. The immunosuppressive schedule included tacrolimus in 54 patients (61.4%) and cyclosporine in 21 (23.9%). Inflammatory skin conditions were diagnosed in 16.8% (21 of 119) of the patients treated with mTOR-Is and in 13.8% (67 of 489) of the patients that were subject to treatment regimens without mTOR-Is. These differences did not achieve a statistical significance.

Infective skin diseases were observed in 29 out of 88 patients (33%), of which 12 were viruses (HPV, VZV, HSV 1 and 2), nine were cutaneous mycoses (*Tinea unguium*, *pytiriasis versicolor* and *tinea corporis*), and five were bacterial infections (folliculitis and impetigo); three patients had concomitant viral and fungal infections.

Clinical details of the three groups of patients can be described as follow.

#### 2.1.1. Psoriasis

**Figure 1:**
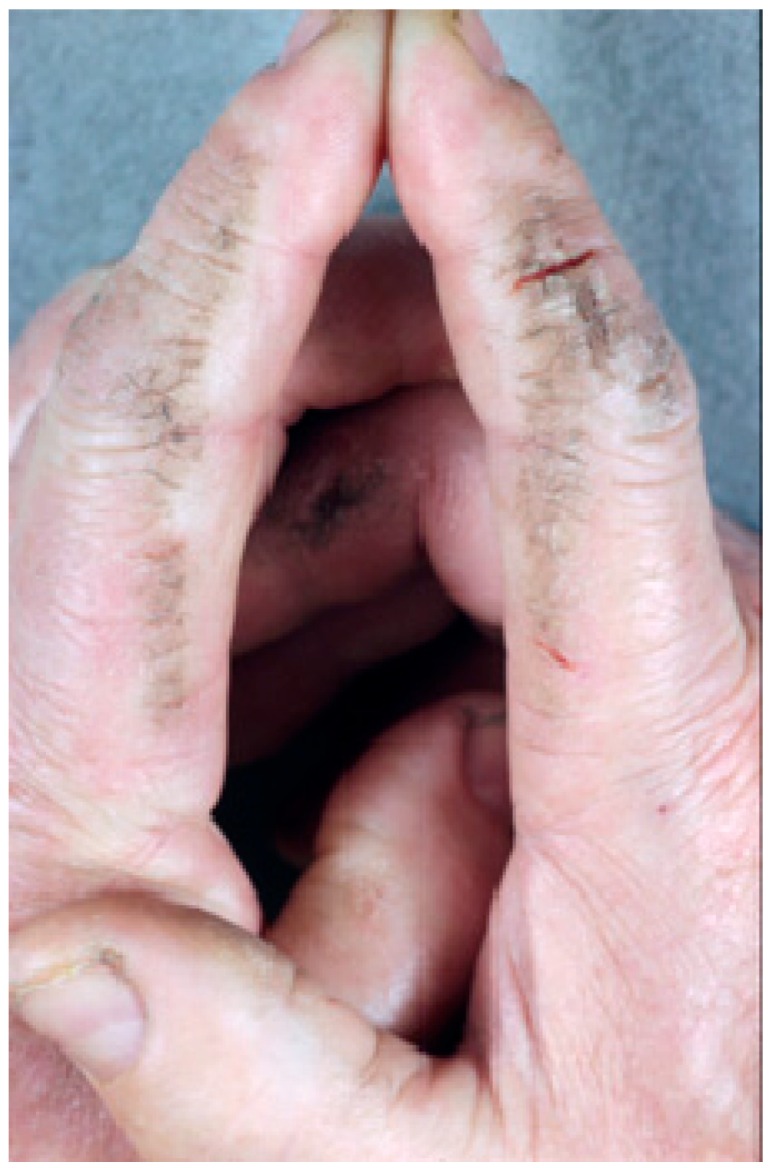
Minimal psoriasis in renal transplant recipient.

We detected psoriasis-related skin conditions in a total of 14 out of 610 patients (2.3%). In all cases, diagnosis was made before the onset of the renal failure. Psoriasis in renal transplant recipients was characterized by a minimal skin involvement (Psoriasis Area Severity Index—PASI score < 3) (Figure 1) and did not require active treatment in addition to the on-going immunosuppressive treatment. The only added treatment was the use of topical emollients in about 30% of cases.

Notably, the skin areas usually affected were the elbow, knee and scalp regions, whereas the involvement of other cutaneous areas or the presence of plaque lesions was extremely rare. No cases of pustular, palmo-plantar or generalized psoriasis were scored.

In two cases of our series, patients reported a clinical improvement of psoriatic lesions after the beginning of the immunosuppressive treatment, in comparison to the pre-transplant period.

#### 2.1.2. Atopic Dermatitis and Related Skin Conditions

**Figure 2:**
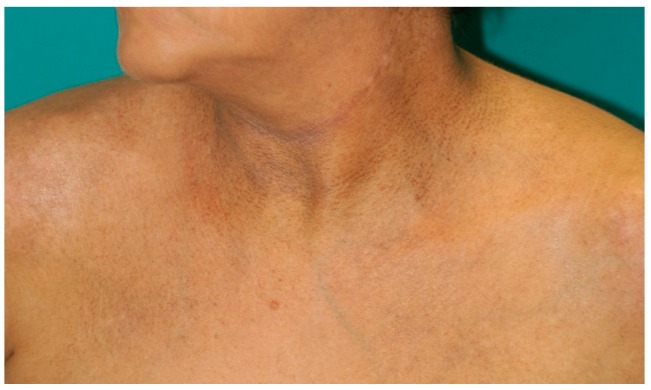
Atopic signs in renal transplant recipient.

**Figure 3:**
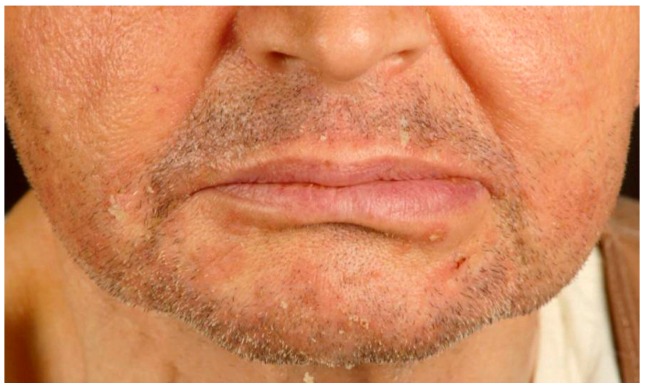
Seborrheic dermatitis in renal transplant recipient.

Atopic lesions were observed in 43 out of 610 cases (7.1%) (Figure 2). Atopic dermatitis was detected in an extremely low percentage of renal transplant recipients, i.e., three cases out of 610 (0.5%). Other atopic-related skin conditions (such as seborrheic dermatitis, nummular eczema, and allergic contact dermatitis) were found in 40 of 610 cases (6.6%). Seborrheic dermatitis (Figure 3) was the most frequent atopic-related skin disorder with a total of 37 cases (86%).

In the majority of patients with atopic and atopic-related skin conditions, emollients were the only topical treatment proposed. No patients required an adjustment of the immunosuppressive treatment to control atopic-related skin conditions.

#### 2.1.3. Other Inflammatory Skin Conditions

**Figure 4:**
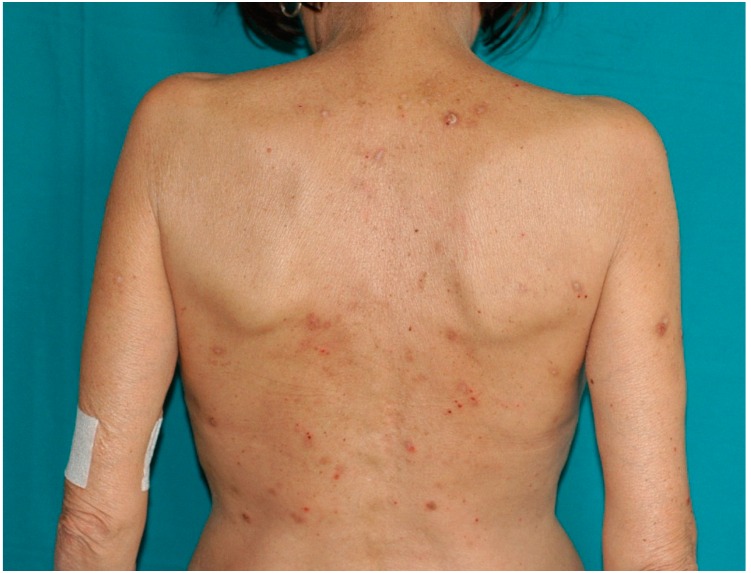
Prurigo nodularis in renal transplant recipient.

Beside atopic dermatitis or psoriasis, 31 patients out of 610 cases (5%) developed other dermatitis. Among these, the most common condition reported was prurigo nodularis (45.2%) (Figure 4), followed by minor aphtosis (20.7%). The other inflammatory conditions detected in our series were reported in Table 3.

Statistical analyses did not identify clinical and/or epidemiological features significantly associated with the development of inflammatory skin disease.

Of note, in our group of patients, 24 (27.3%) reported a skin cancer (11 BCCs, nine SCCs, two melanomas, two Kaposi Sarcomas) after transplantation. No differences in the prevalence of skin cancers were found in the three sub-groups of patients.

## 3. Discussion

Numerous studies have pointed out that skin infections and Non Melanoma Skin Cancers (NMSCs) are commonly occurring complications for transplant recipients [6,8,11,12,13,14] due to the long-term immunosuppression used to prevent transplant rejection. Conversely, scant clinical data are available regarding the incidence of inflammatory skin diseases in patients with solid organ transplantation (Table 4). In a previous study performed to evaluate the incidence of cutaneous diseases in a group of 282 kidney transplant recipients, we reported that inflammatory conditions occur in 14.9% of patients [14], in agreement with previous studies [6,7]; an even lower occurrence of inflammatory diseases in transplanted patients was scored by [8], with only a few cases of acneiform eruptions, rosacea, asteatotic eczema, contact eczema and stasis dermatitis. Notably, the incidence of skin diseases with an immunologic pathogenesis is considered to be an even more uncommon event, with only a few sporadic cases reported [14,15]. Many immunosuppressive drugs—such as tacrolimus, cyclosporine, and mycophenolate—are approved both for preventing chronic transplant rejection and for the treatment of inflammatory skin diseases [9,15,16,17]; this dual therapeutic role can easily explain the observed reduced incidence of inflammatory skin diseases in transplant recipients. In the present study, we observed inflammatory skin diseases in less than 15% of our kidney transplant recipients, versus a more than 60% incidence in the general population [14]. We were not able to identify specific clinical or epidemiological characteristics in patients with inflammatory dermatological diseases, with the exception of a prevalence of males, which, however, is in agreement with the general characteristics of our cohort.

The most common inflammatory disease scored in our transplanted patients was seborrheic dermatitis, which affected a total of 37 patients, accounting for 86% of those with atopic-related skin diseases. This finding is consistent with a previous study [6], but in apparent contrast with the fact that most patients of our series are receiving tacrolimus, commonly used to manage seborrhoeic dermatosis [28]. A possible explanation for this paradoxical behavior could be that only a percentage of seborrhoeic patients are full responders to tacrolimus [28], similarly to those affected by psoriasis [27].

In addition, despite tacrolimus activity in the treatment of atopic dermatitis, a number of atopic dermatitis cases in children with solid organ transplantation have been recently observed: (i) Bumbacea and Ghiordanescu [24] described a six-year old patient who developed a “de novo” atopic dermatitis during long-term immunosuppression with tacrolimus following liver transplantation; (ii) Machura et al. [27] reported a similar case in a three-year-old boy treated with tacrolimus and mycofenolate after heart transplantation. The pathogenesis of this post-transplantation condition is not completely understood and probably involves several factors, including a tacrolimus-induced increase in intestinal permeability, facilitating the absorption of potential allergens and promoting the development of allergy [27]. In our case study, atopic dermatitis was detected in an extremely low percentage of renal transplant recipients, i.e., in three cases out of 610 (0.5%). This can be explained on the basis of the characteristics of our cohort, which was only composed of adult subjects: it is well known that the majority of transplanted patients with atopic dermatitis are children and that a significant risk factor for atopic disease during the post-transplant period is represented by the age of the donor and/or recipient [29]; moreover, allergies are less common described after renal transplantation [30,31].

We also observed a very low percentage of patients affected by psoriasis. The low PASI score confirms that, in the majority of cases, no specific treatment was required in addition to the immunosuppressive regimen to manage psoriatic symptoms. This is possibly due to the high efficacy of calcineurin inhibitors in suppressing psoriasis: we actually observed tacrolimus and cyclosporine efficacy in 61.4% and 23.9% of kidney transplant recipients, respectively. Actually, the therapeutic value of tacrolimus in the treatment of psoriasis was firstly described in transplanted patients [32] and then confirmed in randomized trials [33,34]. Similarly, the effectiveness of cyclosporine in psoriasis has been observed in immunocompetent [34,35] as well as in transplanted patients [22]. Calcineurin inhibitors block the transcription of genes controlling the expression of cytokines, primarily IL-2, and also exert a negative action on regulatory T cell activation (CD4+CD25+FOXp3) [36]. However, a small subset of transplanted patients—especially those who have had a liver transplant—has severe psoriasis that does not fully respond to immunosuppression [22,37,38]. This could be explained by the fact that common immunosuppressive regimens do not completely inhibit all the inflammatory pathways of this pathologic condition, especially the TNF-α and the IL17/23 pathways [24]

Moreover, some authors reported a possible role of mTOR-Is in the pathogenesis of inflammatory skin lesions in kidney transplant recipients [21,29,30,31,36]. In our series, 119 of 610 (19.5%) patients were being treatment by mTOR-Is. Among these patients, the incidence of inflammatory skin conditions was 16.8%, whereas in patients treated with immunosuppressive regiments without mTOR-Is this percentage was 13.8%; even if a trend could be hypothesized, this difference did not achieve a statistical significance.

## 4. Materials and Methods

### 4.1. Patients

Data about 610 renal transplant recipients with a dermatological follow-up at our centers were recorded from January 2009 to April 2016. Sixty-two percent of these patients were males, while 38% were females; median age at transplantation was 51 years and the median duration of immunosuppression was 9.1 years. The median follow-up duration was nine years. For each patient, we evaluated the presence of inflammatory dermatological diseases, which were classified in the following three categories: (i) psoriasis; (ii) atopic dermatitis and related skin conditions (including seborrheic dermatitis, allergic contact dermatitis, and nummular eczema); (iii) other inflammatory dermatitis, unrelated to psoriatic as well as to atopic conditions.

### 4.2. Statistical Analysis

Statistical analysis was performed by IBM SPSS Statistics software (IBM Corp., Armonk, NY, USA) and Kaplan-Meier curves (MedCalc Software, Ostend, Belgium).

## 5. Conclusions

In conclusion, our work emphasizes the low incidence of skin diseases with autoimmune or inflammatory pathogenesis in solid organ-transplanted patients, correlating the beneficial therapeutic effect of these immunosuppressive regimens to the various types of skin disorders.

## Figures and Tables

**Table 1:** Incidence of cutaneous diseases in a cohort of kidney transplant recipients.

Variable	Number	%
Gender		
Male	380	62%
Female	230	37.7%
Immunosuppressive treatment		
Including mTOR-Is	119	19.5%
Without mTOR-Is	491	81.5%
Skin infections	187	30.7%
Viruses	72	
Mycoses	57	
Bacterial	28	
Mixed	30	
Skin cancer	191	31%
Basal Cell Carcinoma (BCC)	94	
Squamous Cell Carcinoma (SCC)	51	
Melanoma	16	
Kaposi’s Sarcoma	17	
Cutaneous T cell lymphoma (CTCL)	4	
others	9	
Inflammatory diseases	88	14.4%

**Table 2:** Clinical characteristics of kidney transplant recipients affected by inflammatory skin diseases.

Variable	Number	%
Gender		
Male	62	70.5%
Female	26	29.5%
Median age	47 years	
Median immunosuppression	9.1 years	
Concomitant diagnosis		
Skin infections	29	33%
Viruses	12	
Mycoses	9	
Bacterial	5	
Mixed	3	
Skin cancer	24	27.3%
BCC	11	
SCC	9	
Melanoma	2	
Kaposi’s Sarcoma	2	

**Table 3:** Other inflammatory conditions detected in our series.

Inflammatory Skin Disease	Number of Patients (%)	Treatment
Prurigo nodularis	14 (45.2%)	Topical
Minor aftosis	6 (19.4%)	Topical
Erythema nodosum	4 (12.8%)	Topical/immunosuppressive treatment adjustment
Zoon balanitis	3 (9.6%)	Topical
Bullous pemfigoid	2 (6.5%)	Topical/immunosuppressive treatment adjustment
Vitiligo	2 (6.5%)	Topical

**Table 4:** Inflammatory cutaneous diseases in solid organ transplant recipients (literature data).

Reference	Clinical Characteristics	Median Follow-up	Cutaneous Disease	Number of Cases
Coehn et al., 1986 [11]	580/kidney	12.2 years	Inflammatory dermatoses	32 (5.5%)
Hoover et al., 2007 [18]	1/liver; Male	Not Available	Psoriasis	1
Kaaroud et al., 2007 [19]	1/kidney; Female 31-year-old	31 months	Pustular psoriasis	1
Collazo et al., 2008 [20]	1/liver; Male 49-year-old	Not Available	Psoriasis	1
Brokalaki et al., 2009 [21]	1/ kidney + pancreas	7 years	Psoriasis	1
	Male 42-year-old			
Wisgerhof et al., 2009 [12]	2136/kidney + pancreas	10.2 years	Psoriasis	4 (0.2%)
			Atopic-related dermatitis	58 (2.7%)
			Others	56 (2.6%)
Lally et al., 2010 [6]	308/kidney; median age 51 years	10.7 years	Psoriasis	5 (1.6%)
			Atopic dermatitis	4 (1.3%)
			Seborrhoeic dermatitis	29 (9.4%)
Saalman et al., 2010 [22]	liver	Not Available	Orofacial granulomatosis	8
Savoia et al., 2011 [14]	282/kidney; median age 59 years	7.2 years	Psoriasis	17 (6%)
			Atopic-related dermatitis	25 (8.8%)
Shroff et al., 2012 [23]	176/liver; median age 16 mo	19 months	Atopic dermatitis	24 (13.6%)
Bumbacea et al., 2013 [24]	1/liver; Male six-year-old	2 years	Atopic dermatitis	1
Moretti de Lima et al., 2013 [8]	53/kidney; median age 44 year	>5 years (52.8%)	Atopic-related dermatitis	4 (7.5%)
			Others	7 (13.2%)
Foroncewicz et al., 2014 [25]	591/liver; median age 50 year	8.5 years	Psoriasis	10 (1.6%)
Machura et al., 2015 [26]	1/heart; Male three-year-old	2.5 years	Atopic dermatitis	1
Madankumar et al., 2015 [27]	1/liver; Female 52-year-old	5 years	Psoriasis	1
